# Exosomes derived from TNF-α-treated bone marrow mesenchymal stem cells ameliorate myocardial infarction injury in mice

**DOI:** 10.1080/15476278.2024.2356341

**Published:** 2024-05-20

**Authors:** Shuo Wang, Rubin Wu, Qincong Chen, Tao Liu, Liu Li

**Affiliations:** a Department of Cardiovascular Medicine, Hebei Medical University of Shijiazhuang People’s Hospital, Shijiazhuang, Hebei, China; b Department of Cardiovascular Medicine, Hebei Medical University Second Hospital, Shijiazhuang, Hebei, China; c Department of Cardiovascular Medicine, Hebei Medical University First Hospital, Shijiazhuang, Hebei, China

**Keywords:** BMSCs, exosomes, macrophage polarization, myocardial infarction, TNF-α

## Abstract

Exosomes derived from bone marrow mesenchymal stem cells (BMSCs) exhibit considerable therapeutic potential for myocardial regeneration. In our investigation, we delved into their impact on various aspects of myocardial infarction (MI), including cardiac function, tissue damage, inflammation, and macrophage polarization in a murine model. We meticulously isolated the exosomes from TNF-α-treated BMSCs and evaluated their therapeutic efficacy in a mouse MI model induced by coronary artery ligation surgery. Our comprehensive analysis, incorporating ultrasound, serum assessment, Western blot, and qRT-PCR, revealed that exosomes from TNF-α-treated BMSCs demonstrated significant therapeutic potential in reducing MI-induced injury. Treatment with these exosomes resulted in improved cardiac function, reduced infarct area, and increased left ventricular wall thickness in MI mice. On a mechanistic level, exosome treatment fostered M2 macrophage polarization while concurrently suppressing M1 polarization. Hence, exosomes derived from TNF-α-treated BMSCs emerge as a promising therapeutic strategy for alleviating MI injury in a mouse model.

## Introduction

Myocardial infarction (MI) is a severe condition characterized by blocked blood supply to the heart, resulting in tissue damage and cell death.^1^ The global prevalence of MI was estimated to be 4.7%-6.4% among adults aged 18 years and older.^2,3^ The prevalence was higher in men than in women and increased with age.^2^ Despite advances in reperfusion strategies, the limited regenerative capacity of the adult heart remains a challenge for effective cardiac repair.^4^

Mesenchymal stem cells (MSCs), especially bone marrow MSCs (BMSCs), have shown promise for myocardial regeneration due to their low immunogenicity and differentiation potential.^5–7^ BMSCs are one of the most widely studied sources of stem cells for MI treatment, because they have low immunogenicity, higher differentiation ability toward to cardiomyocyte-like cells and endothelial cells, which are essential for cardiac function and vascularization.^8–10^ However, it is still controversial for the effectiveness of MSCs therapies in the MI treatment due to the limited cell numbers acquired for the treatment, low efficiency, as well as the delicate route and timing of the injection of the cells. Thus, it would be an alternative option to develop MSCs-based therapies for MI treatment.

In recent years, exosomes, small extracellular vehicles secreted by various cell types, have gained significant attention as mediators of intercellular communication and as potential therapeutic agents.^11^ Exosomes derived from MSCs have been shown to possess regenerative properties and exhibit protective effects in various disease models, including myocardial infarction. ,^12–16^ Compared to cell-based therapies using MSCs, exosome-based therapy offers several advantages, including high efficiency, low immunogenicity, and ease of long-term storage at low temperatures.^17^ Furthermore, exosomes derived from MSCs from different sources exhibit a high degree of consistency in their basic morphological and phenotypic characteristics, as well as their ability to repair acute injuries and prevent fibrosis.^18,19^ These studies suggest it may be more effective using MSCs-derived exosomes instead of MSCs for MI treatment.

Composition and content of exosomes secreted by the same MSCs can significantly vary under different physiological and pathological conditions, and even exhibit contradictory functions.^20,21^ The paracrine effect of MSCs is significantly enhanced by lipopolysaccharide (LPS) pretreatment, leading to the upregulation of anti-inflammatory cytokines and the promotion of M2 macrophage activation.^22,23^ Notably, exosomes released by MSCs in response to inflammatory cytokines have been demonstrated to regulate macrophage polarization, which is crucial for myocardial infarction repair.^24,25^ These findings demonstrate that the anti-inflammatory activities of these exosomes could be enhanced if derived from the cytokine-pretreated MSCs, which may further promote their ability for the MI treatment. In this study, we investigated the impact of TNF-α stimulation on BMSCs in enhancing therapeutic potential of exosomes derived from these cells for myocardial infarction repair. Additionally, we evaluated the effects of these exosomes on macrophage polarization within the microenvironment of myocardial infarction. Our findings contribute to understanding the potential of TNF-α-treated BMSC-derived exosomes as a therapeutic approach for myocardial infarction.

## Materials and methods

### Animals

Twelve-week-old male C57BL/6 mice were procured from GemPharmatech (Nanjing, China). The mice were housed in a controlled laboratory environment, maintaining a temperature of 22 ± 1°C, humidity ranging between 45% and 55%, and a 12-hour light/12-hour dark cycle. The mice were provided with ad libitum access to food and water throughout the study period. All experimental protocols involving animal subjects were approved by the Ethics Committee of Hebei Medical University of Shijiazhuang People’s Hospital (#2023.09.152).

### BMSCs isolation

BMSCs were isolated from the bone marrow of C57BL/6 mice following a standardized protocol.^26^ In brief, the bone marrow was collected by flushing the femurs and tibias of the mice using a 2% heat-inactivated fetal bovine serum (FBS, Gibco, Grand Island, NY) solution. The collected bone marrow cells were then processed to isolate mononuclear cells (MNCs) using density gradient centrifugation. Subsequently, the MNCs were further purified by isolating mononuclear granulocytes through the utilization of CD11b^+^ microbeads (StemCell Technologies, Vancouver, Canada). The remaining cells were subjected to culture conditions, by supplementing Minimum Essential Medium α (MEM α, ThermoFisher, Waltham, MA) with 10% FBS and 1% of Penicillin/Streptomycin, at 37°C in an incubator with 5% CO_2_. Following incubation for 72–96 hours, non-adherent cells were carefully removed, while the adherent BMSCs were maintained and cultured until reaching approximately 75% confluence. These cultured BMSCs were subsequently used for the following experimental procedures.

### TNF-α stimulation of BMSCs

Recombinant human TNF-α was procured from Biolegend (San Diego, CA). BMSCs (1.5 × 10^6^ cells/well) at the third passage were subjected to two distinct culture conditions: with or without the addition of 20 ng/ml TNF-α in the 6-well plate. The cells were incubated under these respective conditions for a duration of 48 hours as described previously.^15^

### Exosome isolation

Isolation of BMSCs-derived exosomes followed a previously described method.^15^ Briefly, the supernatants from BMSC cultures were collected and subjected to a series of centrifugation steps: first, the supernatants were centrifuged at 500 g for 10 minutes to remove cellular debris. The resulting supernatants were then collected and subjected to a second centrifugation at 12,000 g for 20 minutes to pellet larger vesicles. Subsequently, the supernatants were collected again and subjected to ultracentrifugation at 100,000 g for 2 hours to obtain exosome pellets. The isolated exosomes were resuspended in phosphate-buffered saline (PBS) for subsequent analysis. The size distribution of the exosomes was determined using Nanoparticle Tracking Analysis (Malvern Panalytical, Malvern, UK). Exosome identification and profiling reveal that there is no discernible difference in exosome concentration and size between TNF-α-treated BMSCs and their non-treated counterparts.

### MI model and treatment

To induce MI, 12-week-old C57BL/6 mice were anesthetized and positioned on an operating table.^27^ Mice were connected to a respirator to ensure proper ventilation throughout the procedure. Surgical exposure was achieved by beveling the third and fourth ribs, allowing visualization of the hearts. The coronary artery was then ligated using 6–0 nylon thread with three knots. Immediate paleness in the lower left region of the left ventricle confirmed successful ligation. Subsequently, 5 μg of exosomes suspended in 25 μL of PBS were intramyocardially injected at five distinct points surrounding the MI area, following a previously established protocol.^23^ The incision was carefully sutured, cleaned, and disinfected after the injection. Samples for analysis were collected after a 28-day period following the induction of MI.

### Cardiac function analysis

Cardiac function of mice with indicated treatment was detected by using Ruihua ultrasound instrument (Xuzhou, China), including of left ventricular ejection fraction (LVEF), left ventricular fractional shortening (LVFS), left ventricular internal dimension at diastole (LVIDd), and left ventricular internal dimension at systole (LVIDs), as described previously.^28^

### Quantitative reverse transcription PCR (qRT-PCR)

Total RNA was extracted from the heart tissues using TRIzol (Invitrogen, Waltham, MA) and then transcribed to cDNA by using superscript III reverse transcriptase and random primers following the manufacturer's instruction (Invitrogen). Target genes expression levels were determined through SYBR Green PCR Kit using ProFlex Flat PCR System (ThermoFisher). The primers used were as follows (5’-3’):

#### Cd86

Forward: TCAATGGGACTGCATATCTGCC,

Reverse: GCCAAAATACTACCAGCTCACT;

#### Cd206

Forward: GGGACTCTGGATTGGACTCA,

Reverse: GCTCTTTCCAGGCTCTGATG;

#### Gapdh

Forward: AATGGATTTGGACGCATTGGT,

Reverse: TTTGCACTGGTACGTGTTGAT.

### Histological and immunofluorescent assays

Hearts were collected at 28 days post-MI, fixed in 4% paraformaldehyde overnight, and subsequently embedded in paraffin. Tissue sections of 5 μm thickness were obtained and subjected to various staining techniques for quantitative analysis. Hematoxylin and eosin staining (H&E) was performed to assess inflammatory cell infiltration. Immunofluorescence staining using anti-CD206 F4/80, and iNOS primary antibodies (1:500 dilution, Abcam, ab178945), and CD206 (1:500 dilution, CST, #24595), F4/80 (1:500 dilution, CST, #70076) were performed to quantify M2 macrophage polarization in the myocardial tissues after MI in mice.^29^ Image quantification of the sections was carried out using ImageJ.

### Western blot

Protein extraction from exosomes performed using radioimmunoprecipitation assay buffer supplemented with protease inhibitors (Sigma-Aldrich, St. Louis, MO). The protein samples were separated by 10% sodium dodecyl sulfate – polyacrylamide gel electrophoresis (Beyotime, Shanghai, China) and subsequently transferred onto PVDF membranes (Life Technology, Waltham, MA). After blocking with 5% skim milk at room temperature for 1 hour, the membranes were incubated overnight at 4°C with the appropriate primary antibodies, CD63 (1:1000 dilution, CST, #52090), CD9 (1:500 dilution, CST, #98327), and Alix (1:1000 dilution, CST, #92880). Following the primary antibody incubation, the membranes were incubated with the corresponding secondary antibody for an additional 1 hour at room temperature. Protein bands were visualized using an ECL chemiluminescence kit (Vazyme, Nanjing, China) according to the manufacturer’s instructions.

### Statistical analysis

Statistical analysis was performed on the collected data using appropriate methods. All data, unless specified, were included in the analysis. The results are presented as the mean ± standard deviation (SD). Statistical significance was determined using Brown-Forsythe ANOVA test followed by Dunnett’s T3 multiple comparisons test. A p-value less than 0.05 was considered statistically significant. All statistical analyses were conducted using Prism 8.0.

## Results

### Exosomes derived from TNF-α-treated BMSCs attenuated cardiac injury after MI in mice

**Figure 1.**
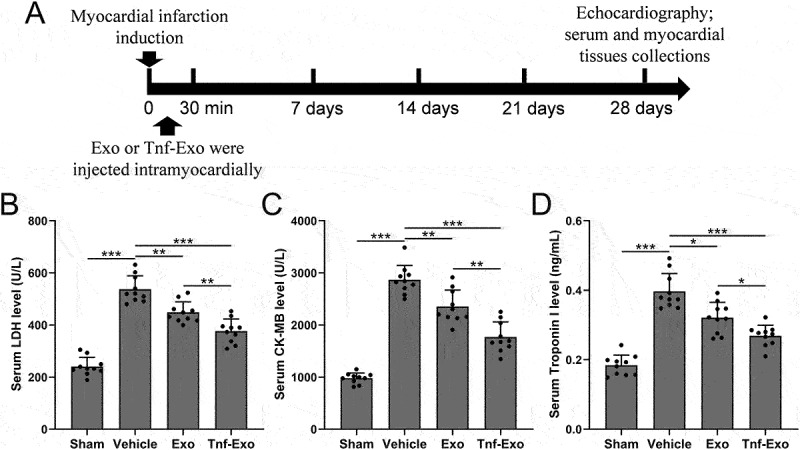
Attenuation of cardiac injury by exosomes from TNF-α-treated BMSCs in the MI mouse model.

**Figure 2.**
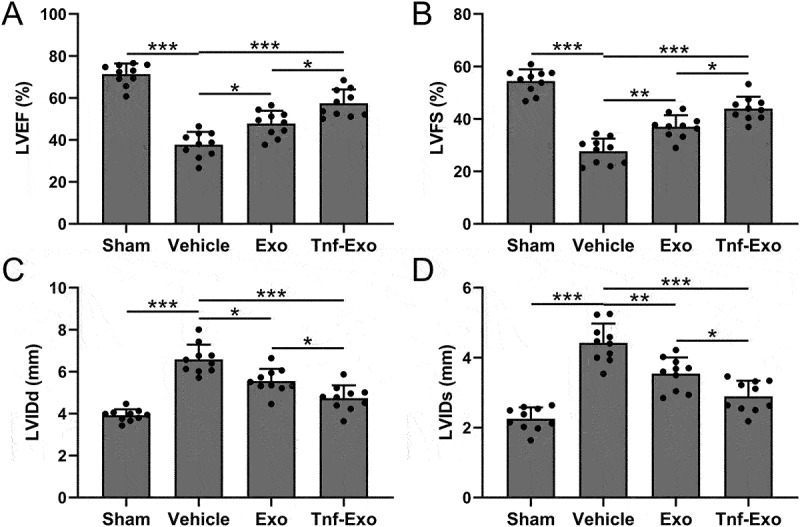
Preservation of cardiac function by exosomes from TNF-α-treated BMSCs in the MI mouse model.

The experimental design commenced with the induction of MI in mice on day 0 through surgical procedures involving ligation of the left anterior descending branch. Subsequently, mice were intramyocardially injected with transfected exosomes (5 μg in 25 μL PBS) at five points surrounding the MI region, within 30 minutes of the surgery (Figure 1a). On day 28 following MI, relevant assessments were conducted. Initially, the levels of cardiac injury biomarkers, including serum lactate dehydrogenase (LDH) (Figure 1b), creatine kinase-MB (CK-MB) (Figure 1c), and Troponin I (Figure 1d), were compared. The results demonstrated that treatment with exosomes effectively reduced the levels of cardiac injury biomarkers in the serum (Figure 2b-d). Furthermore, exosomes derived from TNF-α-treated BMSCs exhibited a more significant therapeutic effect. These data suggested that TNF-α-treated BMSCs-derived exosomes could attenuate MI-induced cardiac injury in mice.

(a) Study design of animal experiments to investigate the impact of exosomes derived from TNF-α-treated BMSCs on cardiac injury post-MI. At 28 days post-MI, serum levels of key cardiac injury markers, including LDH (Lactate Dehydrogenase) (b), CK-MB (Creatine Kinase-MB) (c), and Troponin I (d), were quantified. These analyses served as pivotal indicators in assessing the therapeutic efficacy of the administered exosomes. Data were shown with mean ± SD. 10 mice were used for each group. *p < .05, **p < .01, ***p < .001 from Brown-Forsythe ANOVA test followed by Dunnett’s T3 multiple comparisons test.

The protective effects of exosomes derived from TNF-α-treated BMSCs on cardiac function post-MI is evidenced through comprehensive assessments of key parameters, including Left Ventricular Ejection Fraction (LVEF) (a), Left Ventricular Fractional Shortening (LVFS) (b), Left Ventricular Internal Diameter at end-diastole (LVIDd) (c), and Left Ventricular Internal Diameter at end-systole (LVIDs) (d) at the 28-day mark post-MI. These comparisons elucidate the therapeutic impact of the administered exosomes on preserving overall cardiac function. Data were shown with mean ± SD. 10 mice were used for each group. *p < .05, **p < .01, ***p < .001 from Brown-Forsythe ANOVA test followed by Dunnett’s T3 multiple comparisons test.

### Exosomes derived from TNF-α-treated BMSCs protected cardiac function after MI in mice

Next, we used the small animal echocardiography to analyze the cardiac function of mice in different treatment groups, including LVEF (Figure 2a), LVFS (Figure 2b), LVIDd (Figure 2c), LVIDs (Figure 2d). In comparison with vehicle control group, mice in Tnf-Exo treated had significant improvement of both LVEF and LVFS, accordingly, the LVIDd and LVIDs were remarkedly reduced after Exo and Tnf-Exo treatment (Figure 2a-d). The results revealed that treatment with exosomes effectively improved the deterioration of cardiac function in mice after MI. Importantly, exosomes derived from TNF-α-treated BMSCs exhibited a more pronounced therapeutic effect.

### Exosomes derived from TNF-α-treated BMSCs alleviated infarct size after MI in mice

**Figure 3.**
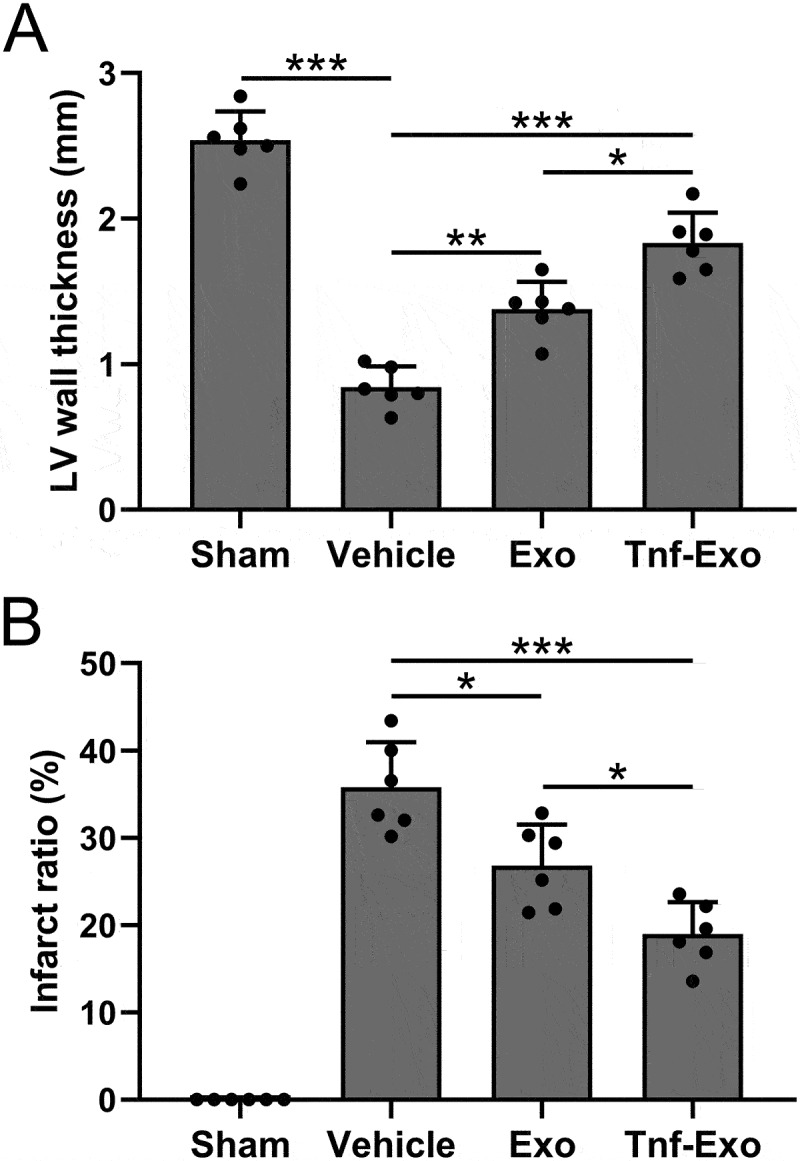
Exosomes derived from TNF-α-treated BMSCs alleviated infarct size after myocardial infarction in mice.

Subsequently, we performed H&E staining to analyze the left ventricular wall thickness and infarct area in mice from different groups after 28 days of MI. The results demonstrated that treatment with exosomes effectively and increased the thickness of the left ventricular wall (Figure 3a) and reduced the extent of the infarct area (Figure 3b). Notably, exosomes derived from TNF-α-treated BMSCs exhibited a more pronounced therapeutic effect (Figure 3a,b). The above data indicated that TNF-α-treated BMSCs-derived exosomes could alleviate infarct size in MI-induced cardiac injury mice.

(a) The calculations of left ventricular wall thickness and (b) infarct ratio in the experimental groups. Data were shown with mean ± SD. 6 mice were used for each group. *p < .05, **p < .01, ***p < .001 from Brown-Forsythe ANOVA test followed by Dunnett’s T3 multiple comparisons test.

### Exosomes derived from TNF-α-treated BMSCs enhanced M2 macrophage polarization in the myocardial tissues after MI in mice

**Figure 4.**
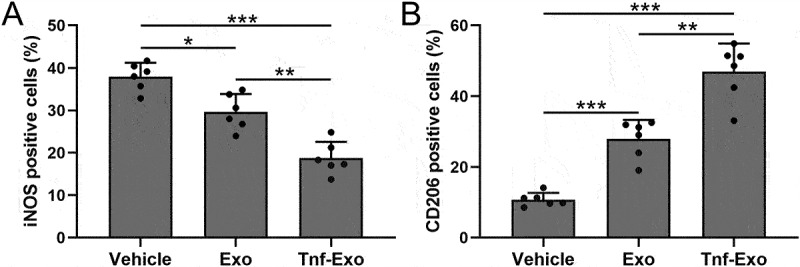
Exosomes derived from TNF-α-treated BMSCs enhanced M2 macrophage polarization in the myocardial tissues after myocardial infarction in mice.

Next, we analyzed the polarization of macrophages in the infarct area of mice from each group after 28 days of MI. CD206 is a marker of M2 macrophage polarization, while iNOS is a marker of M1 polarization.^28,30^ The results showed that MI led to the activation of macrophages in the infarcted tissue of mice, with an excessive imbalance between M1 and M2 polarization (Figure 4a,b). However, treatment with exosomes effectively promoted M2 polarization and suppressed M1 polarization (Figure 4a,b). Notably, exosomes derived from TNF-α-treated BMSCs exhibited a more pronounced therapeutic effect (Figure 4a,b).

The percentage of M1 (a) and M2 (b) polarized macrophages in the total number of macrophages in the infarct area of myocardial tissues at 28 days post-MI. Data were shown with mean ± SD. 6 mice were used for each group. *p < .05, **p < .01, ***p < .001 from Brown-Forsythe ANOVA test followed by Dunnett’s T3 multiple comparisons test.

### Exosomes derived from TNF-α-treated BMSCs enhanced M2 macrophage polarization in the myocardial tissues after MI in mice

**Figure 5.**
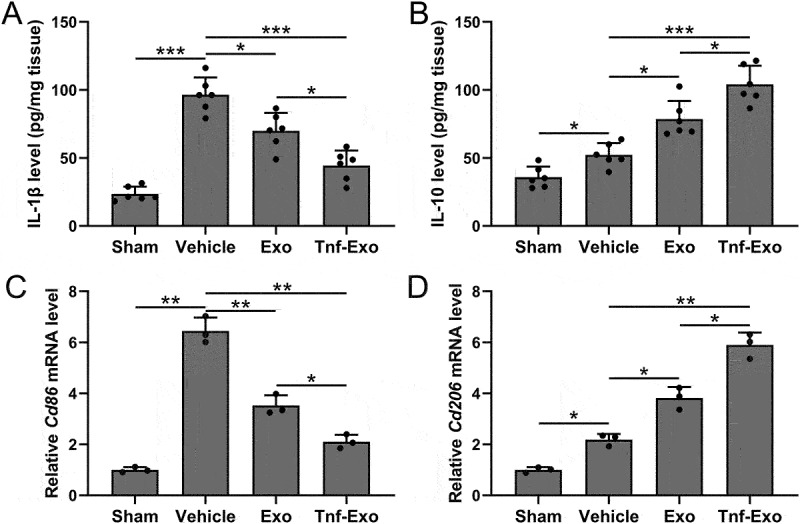
Exosomes derived from TNF-α-treated BMSCs enhanced M2 macrophage polarization in the myocardial tissues after myocardial infarction in mice.

We examined the protein levels of the pro-inflammatory cytokine IL-1β, associated with M1 polarization, and the anti-inflammatory cytokine IL-10, associated with M2 polarization, in the infarcted area of mice. The results of our analysis further confirmed that treatment with exosomes effectively promoted M2 polarization while suppressing M1 polarization (Figure 5a,b). Moreover, exosomes derived from TNF-α treated BMSCs exhibited a more significant therapeutic effect (Figure 5a,b). The mRNA expression levels of Cd86 and Cd206, markers of M1 and M2 polarization, respectively, in the infarcted area of mice, provided additional support for the enhanced M2 macrophage polarization by exosomes derived from TNF-α-treated BMSCs in the myocardial tissues following myocardial infarction (Figure 5c,d). These results suggested that TNF-α-treated BMSCs-derived exosomes could enhance M2 macrophage polarization in the myocardial tissues of MI-induced cardiac injury mice.

ELISA was used to measure the levels of IL-1β (a) and IL-10 (b) in left ventricular tissues at 28 days post-MI. 6 mice were used for each group. qRT-PCR was used to measure the mRNA expressions of Cd86 (c) and Cd206 (d) in left ventricular tissues at 28 days post-MI. 6 mice were used for each group and the experiments were repeated 3 times using mixed tissue homogenates. Data were shown with mean ± SD. *p < .05, **p < .01, ***p < .001 from Brown-Forsythe ANOVA test followed by Dunnett’s T3 multiple comparisons test.

## Discussion

MI poses a formidable challenge due to its detrimental impact on cardiac function, primarily attributed to the loss of viable cardiomyocytes.^1^ Despite ongoing therapeutic explorations, there remains an imperative for effective strategies to enhance myocardial repair and functional recovery.^2,4^ In this study, we explored the potential therapeutic role of exosomes derived from TNF-α-treated BMSCs in ameliorating MI injury.

Exosomes, intricate mediators of intercellular communication, have emerged as promising therapeutic agents in the regenerative landscape.^11,15^ Our results underscored the superior therapeutic efficacy of exosomes from TNF-α-treated BMSCs compared to their untreated counterparts. This observation hints at the modulatory influence of TNF-α treatment on exosomal cargo, heightening their regenerative potential. The multifaceted benefits of exosome treatment were evident on several fronts. First, we observed a significant reduction in myocardial injury markers, including serum levels of LDH, creatine kinase-MB (CK-MB), and troponin I, indicating a protective effect on cardiomyocytes. This finding is consistent with previous studies demonstrating the ability of exosomes to attenuate myocardial damage and preserve cardiac function.^15, 23, 24, 31^ Furthermore, histological analyses revealed that treatment with exosomes led to a decrease in the infarct area and an increase in left ventricular wall thickness. These findings suggest that exosomes contribute to the inhibition of adverse remodeling processes and promote structural preservation of the myocardium.^32,33^ The improvement in cardiac function, as substantiated by echocardiography, further bolsters the therapeutic promise of exosomes in MI.

Macrophage polarization plays a crucial role in the inflammatory response following MI.^34,35^ Excessive M1 polarization and impaired M2 polarization have been associated with adverse cardiac remodeling.^36,37^ Exosome treatment demonstrated a proclivity toward promoting M2 macrophage polarization while suppressing M1 polarization, as evidenced by altered expression levels of markers such as iNOS and CD206. This shift in polarization potentially contributes to inflammation attenuation and tissue repair promotion. Furthermore, examination of pro-inflammatory cytokine IL-1β and anti-inflammatory cytokine IL-10 in the infarcted area revealed a pronounced increase in IL-10 expression and a reduction in IL-1β levels post-exosome treatment. These findings substantiate the anti-inflammatory and immunomodulatory prowess of exosomes in the MI context.

It is imperative to acknowledge certain limitations. The intricate mechanisms through which exosomes from TNF-α-treated BMSCs exert therapeutic effects necessitate further exploration. Detailed investigation into the specific cargo components, such as proteins and microRNAs, could unravel the molecular intricacies underlying their enhanced regenerative properties. Additionally, delineating the signaling pathways involved in mediating these effects remains an avenue for future studies. Despite these limitations, our findings provide compelling evidence supporting the therapeutic potential of TNF-α-treated BMSCs-derived exosomes in mitigating MI injury and warrant further exploration of their intricate molecular mechanisms.

## Conclusion

Our study provides compelling evidence for the therapeutic potential of exosomes derived from TNF-α-treated BMSCs in ameliorating myocardial injury following MI. These exosomes exhibited superior efficacy in preserving cardiac function, reducing myocardial injury, promoting structural preservation, and modulating macrophage polarization. These findings support the notion that exosomes could serve as a promising therapeutic approach for myocardial repair and highlight the importance of TNF-α priming in enhancing the regenerative properties of exosomes derived from BMSCs.

## Data Availability

The raw data supporting the conclusions of this article will be made available by the authors, without undue reservation.

## Abbreviations

MSCs: Mesenchymal stem cells
BMSCs: Bone marrow mesenchymal stem cells
MI: Myocardial infarction
MNCs: Mononuclear cells
MEM α: Minimum Essential Medium α
PBS: Phosphate-buffered saline
FBS: Fetal bovine serum
LVEF: Left ventricular ejection fraction
LVFS: Left ventricular fractional shortening
LVIDd: Left ventricular internal dimension at diastole
LVIDs: Left ventricular internal dimension at systole
qRT-PCR: Quantitative reverse transcription PCR
LDH: Lactate dehydrogenase
CK-MB: Creatine kinase-MB
